# Genetic diversity of male and female Chinese bayberry (*Myrica rubra*) populations and identification of sex-associated markers

**DOI:** 10.1186/s12864-015-1602-5

**Published:** 2015-05-19

**Authors:** Hui-min Jia, Yun Jiao, Guo-yun Wang, Ying-hui Li, Hui-juan Jia, Hong-xia Wu, Chun-yan Chai, Xiao Dong, Yanping Guo, Liping Zhang, Qi-kang Gao, Wei Chen, Li-juan Song, Eric van de Weg, Zhong-shan Gao

**Affiliations:** Department of Horticulture, College of Agriculture and Biotechnology, Zhejiang University, 310058 Hangzhou, Zhejiang China; Fruit Research Institute, 315400 Yuyao, Ningbo PR China; The National Key Facility for Crop Gene Resources and Genetic Improvement (NFCRI)/Key Lab of Germplasm Utilization (MOA), Chinese Academy of Agricultural Sciences, Institute of Crop Science, 100081 Beijing, China; Forestry Technology Extension Center, 315300 Cixi, Ningbo China; Bio-Macromolecules Analysis Lab, Analysis Center of Agrobiology, Environmental Sciences of Zhejiang University, 310058 Hangzhou, China; Zhejiang Institute of Subtropical Crops, Wenzhou, 325005 China; Wenzhou Vocational and Technical College, 325035 Wenzhou, China; Plant Breeding-Wageningen University and Research Centre, P.O. Box 16, 6700 AA Wageningen, The Netherlands

## Abstract

**Background:**

Chinese bayberry (*Myrica rubra* Sieb. & Zucc.) is an important subtropical evergreen fruit tree in southern China. Generally dioecious, the female plants are cultivated for fruit and have been studied extensively, but male plants have received very little attention. Knowledge of males may have a major impact on conservation and genetic improvement as well as on breeding. Using 84 polymorphic SSRs, we genotyped 213 *M. rubra* individuals (99 male individuals, 113 female varieties and 1 monoecious) and compared the difference in genetic diversity between the female and the male populations.

**Results:**

Neighbour-joining cluster analysis separated *M. rubra* from three related species, and the male from female populations within *M. rubra*. By structure analysis, 178 *M. rubra* accessions were assigned to two subpopulations: Male dominated (98) and Female dominated (80). The well-known cultivars ‘Biqi’ and ‘Dongkui’, and the landraces ‘Fenhong’ are derived from three different gene pools. Female population had a slightly higher values of genetic diversity parameters (such as number of alleles and heterozygosity) than the male population, but not significantly different. The SSR loci ZJU062 and ZJU130 showed an empirical Fst value of 0.455 and 0.333, respectively, which are significantly above the 95 % confidence level, indicating that they are outlier loci related to sex separation.

**Conclusion:**

The male and female populations of Chinese bayberry have similar genetic diversity in terms of average number of alleles and level of heterozygosity, but were clearly separated by genetic structure analysis due to two markers associated with sex type, ZJU062 and ZJU130. Zhejiang Province China could be the centre of diversity of *M. rubra* in China, with wide genetic diversity coverage; and the two representative cultivars ‘Biqi’ and ‘Dongkui’, and one landrace ‘Fenhong’ in three female subpopulations. This research provides genetic information on male and female Chinese bayberry and will act as a reference for breeding programs.

**Electronic supplementary material:**

The online version of this article (doi:10.1186/s12864-015-1602-5) contains supplementary material, which is available to authorized users.

## Background

Chinese bayberry (*Myrica rubra*) is an evergreen fruit tree native to southern China and other Asian countries [1]. The fruit has a delicious flavour and high nutritional value, especially rich in anthocyanins. It can be eaten as fresh fruit and as processed products, and has become popular in China and other countries in recent years [2-4]. Among the six *Myrica* species in China, only *M. rubra* is commercially cultivated in Zhejiang, Jiangsu, Fujian and Guangdong, and is also grown in the Yunnan, Guizhou and Hunan provinces [5,6].

**Fig. 1:**
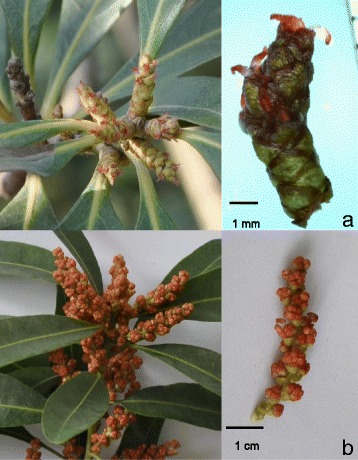
Flower morphology of Chinese bayberry. **a**, female; **b**, male

Chinese bayberry (2n = 16) belongs to the *Myricaceae* family, is usually dioecious, is wind pollinated, and only a few individuals are monoecious. Other species in the Myricaceae family such as *M. cerifera*, *M. faya* and *M. rivas-martinezii* are also dioecious plants [7-9], with unclear mechanism of sex determination. Monoecious plants have also been found in *M. faya* [9]. *M. rubra* has a symbiotic association with nitrogen-fixing bacterium in the root system, which has also been found in other *Myricaceae* family members [7,10-12]. The morphology of its inflorescences and flowers varies with the sex of the tree (Fig. 1). Male plants, with a different colour and shape of staminate catkins, are planted for landscape and pollination purposes [1]. The number of male plants is diminishing year by year because of their low economic benefits. Even though Chinese bayberry was domesticated in southern China more than 2000 years ago, it only has about fifty years of research history. The rich germplasm of Chinese bayberry is reflected by around 300 recorded landraces/cultivars [5], also some landraces including a group rather than a single scion variety. Zhejiang Province has the longest history of Chinese bayberry cultivation, with considerable germplasm resources. The main cultivars ‘Biqi’ (with black fruit and average weight of 11 g) and ‘Dongkui’ (red fruit and average weight of 22 g) are pure scion cultivars, and have been widely cultivated in China. While two landraces ‘Fenhong’ (pink fruit and average weight of 13 g) and ‘Shuijing’ (light yellow fruit and average weight of 15 g) are limited to local regions in Ningbo area of Zhejiang Province. Mutation and natural elite line identification has been the dominant way of breeding new cultivars [6].

Genetic diversity, cultivar identification and the geographic origin of Chinese bayberry have been studied using molecular markers [5,6,13], but based on the dozen available markers [14,15], with the male plants rarely used in the study of cross breeding and genetic diversity. With the development of sequencing techniques, a large number of simple sequence repeat markers (SSRs) have been isolated in recent years [13,16,17]. Using SSR markers, no genetic differentiation has been found between *M. rivas-martinezii* and *M. faya* [8]. SSRs are powerful markers which have been widely used in studies on genetic diversity and population structure, and to construct linkage maps [18-22].

Molecular markers linked to sex determination have been reported in a few dioecious fruit crops. A microsatellite (GATA)n revealed sex-specific differences in papaya [23], and SCAR markers were then developed for sex determination [24,25]. Fraser found that sex-linked SCAR markers and the ‘Flower-sex’ phenotype mapped to the same linkage group in the dioecious species *Actinidia chinensis* (kiwifruit), which revealed putative X/Y sex-determining chromosomes[19]. However, there has been no report on male plant genetic diversity and sex determination in Chinese bayberry or other *Myrica* species. Here we report, for the first time, the genetic diversity of male plants of Chinese bayberry compared with the female plants, using 84 SSR markers. This information will be useful in the conservation of these diverse individuals and for direct application in the new initiative of the Chinese bayberry cross-breeding program.

## Results

### Genetic diversity of the *M. rubra* accessions

The 84 SSRs used in this research were polymorphic in both female and male samples, amplifying a total of 876 alleles, with an average of 10.43 alleles per locus. (Additional file 1: Table S1). The number of alleles per locus (Na) ranged from 3 to 23. The frequency of more than half of the alleles (526, 60.05 %) was less than 5 % and 236 were unique (frequency less than 1 %). Low allele frequencies resulted in a reduced effective number of alleles (Ne, 3.71). The observed heterozygosity (Ho) ranged from 0.11 to 0.90, with an average of 0.49. For most loci (72) the *Ho* value was lower than the expected (He), which ranged from 0.19 to 0.88, (mean, 0.65). Shannon’s information index (*I*) for each locus ranged from 0.47 to 2.36, with a mean of 1.48.

The Na, Ne, Ho, and Hs of the male and female populations in 84 SSR loci are shown in Table 1. Across all 84 SSR loci, we found fewer Na (8.71) in the male population than in the female (9.04). The sample of male accessions (99) was smaller than that of the female population (113 accessions), so to correct for this difference in sample size, the means of distinct and private alleles per locus were analysed as a function of the sample size for the two populations (Additional file 2: Figure S1). With increasing sample size, the number of distinct and private alleles also increased, and it was clear that the female population had a slightly higher value than the male population. Moreover, the female population had a slightly higher mean effective number of alleles (3.60) and Hs (0.65) than the male population (3.34 and 0.62, respectively), although not significant (*t* test, *P* = 0.2530). Apart from the differences between them, these results also illustrate that the female and male of Chinese bayberry have abundant genetic diversity.

**Table 1:** Genetic diversity of Chinese bayberry accession subsets based on 84 SSRs

Subset of accessions	Sample size	*Na*	*Ne*	*Ho*	*Hs*	*F*
All accessions	213	10.11	3.75	0.49	0.65	0.25
Female population	114	9.04	3.60	0.55	0.65	0.34
Male population	99	8.75	3.34	0.40	0.62	0.15

Note: *Na*: number of observed alleles; *Ne:* effective number of alleles; *Ho*: observed heterozygosity; Hs: gene diversity; F: Wright’s fixation index.

### Population Structure

**Fig. 2:**
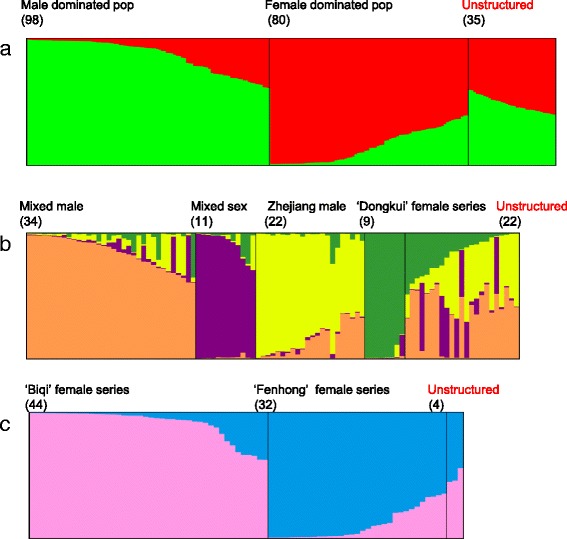
Population stratification based on Bayesian clustering approaches, with a value of k from 1 to 10. Each individual is shown as a thin vertical line, different subpopulations are in a different colour. The name of each population or subpopulation and the number of individuals included is given at the top of each column. **a**, The first STRUCTURE step with 213 accessions, k = 2. The subpopulation was displayed by DISTRUCT, and each subpopulation ordered according to the membership coefficient. **b**, Nest structure analysis for the Male Dominated pop. mainly divided into four subpopulations. **c**, Nest structure analysis for the Female Dominated pop. which was further divided into two subpopulations. Note: Unstructured indicates individuals which were not assigned to any population

The 213 *M. rubra* accessions were evaluated for population stratification. The assumed number of populations was set from *K* = 1 to 10. According to the method of Evanno [26], the accessions were mainly divided into two subpopulations (K = 2). CLUMPP alignment of 10 independent solutions for K = 2 showed pairwise ‘G’ values around 0.99, indicating that the assignment of genotypes to the subpopulations was well correlated between runs. Setting the membership coefficient to Q ≥ 0.6, 178 genotypes could be generally clustered into two subpopulations (Fig. 2a), one the Male Dominated pop. (green in Fig. 2a) with 82 male and 16 female plants, and the other the Female Dominated pop. (red in Fig. 2a) with 76 female and four male plants. The remaining 35 genotypes were unstructured: about half of them (17) were female cultivars from the Hunan, Jiangsu and Guizhou provinces. The monoecious plants (C2010-4) also clustered within this admixed subpopulation.

STRUCTURE was then run for each of the subpopulations as a first step. The Male Dominated population of plants (Fig. 2a, in green) was further divided into four groups (Fig. 2b). The first, the Mixed male subpopulation (orange in Fig. 2b), included 27 male plants from five provinces of south China. The Mixed sex subpopulation (purple in Fig. 2b) included five female plants from Guangdong and Zhejiang and three male plants from Zhejiang and Jiangsu provinces, and the third subpopulation, Zhejiang male (yellow in Fig. 2b), included 21 male plants and one cultivar from Zhejiang Province. In the fourth subpopulation, the ‘Dongkui’ female series, there were seven landraces from Zhejiang, one cultivar (Heiruilin) from Taiwan and one (Ruiguangmei) from Japan.

The Female-dominated population was further structured into two groups (Fig. 2c). The ‘Biqi’ female series was largely restricted to plants from Zhejiang (41), also with two from Fujian and one from the Jiangxi Provinces. Eight of the ten cultivars from Zhejiang were ‘Biqi’-derived cultivars, with three male plants Y2012-1, Y2012-2 and Y2010-16 also in the ‘Biqi’ female series. The ‘Fenhong’ female series included 31 female and one male (‘Y2012-151’) accessions mainly from Zhejiang Province, with seven and eight of the 31 female accessions belonging to the ‘Shuijing’ and ‘Fenhong’ series respectively.

**Fig. 3:**
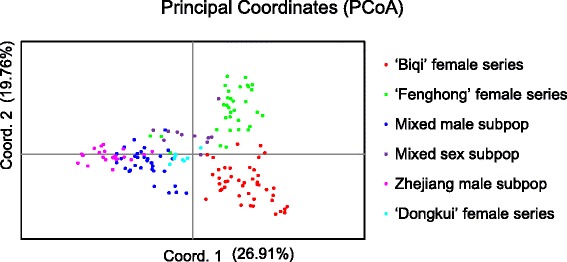
Principal coordinate analysis (PCoA) of 152 *M. rubra* accessions. The different colours represent the six subpopulations inferred by nest STRUCTURE analysis. The first and second principal coordinates account for 26.91 % and 19.76 % of the total variation, respectively

Based on AMOVA analysis, most variation (81.55 %) was detected within individual accessions, with only 11.00 % attributed to variation among the six subpopulations (Table 2). The overall Fst among the six subpopulations was 0.1100 (p value < 0.05). Pairwise Fst values ranged from 0.0728 (between the ‘Zhejiang male’ and ‘Mixed male’ subpopulations) to 0.2346 (between the ‘Zhejiang male’ and ‘Dongkui female’ series) (Table 3). The genetic diversity of 178 structured accessions was also confirmed by PCoA (Fig. 3). The first three axes together accounted for 63.65 % of the variation. The first and second coordinates accounted for 26.91 % and 19.76 % of the molecular variation, respectively, with the first coordinate separating Male Dominated population from Female Dominated population accessions, and the second coordinate separating the ‘Biqi’ and the ‘Fenhong’ female series.

**Table 2:** Analysis of molecular variance (AMOVA) among subpopulations inferred by STRUCTURE analysis, based on the 84 SSR loci of 178 Chinese bayberry accessions

Source of variation	d.f.	Sum of squares	Variance components	Fixation indices	Percentage of variation
Among populations	5	530.026	1.86626 Va	Fst = 0.11005	11.00
Among individuals within subpopulations	146	2387.945	1.26342 Vb	Fis = 0.08371	7.45
Within individuals	152	2102.000	13.82895 Vc	Fit = 0.18455	81.55
Total	303	5019.970	16.95863		

Fis: inbreeding coefficient of subpopulations, Fit: inbreeding coefficient in the total sample, Fst: genetic differentiation among subpopulations.

**Table 3:** Pairwise estimates of Fst among the six subpopulations, based on 84 SSRs

	‘Biqi’ female series	‘Fenhong’ female series	Mixed sex	Zhejiang male	‘Dongkui’ female series	Mixed male
‘Biqi’ female series	0.0000					
‘Fenhong’ female series	0.0945	0.0000				
Mixed sex	0.1118	0.0756	0.0000			
Zhejiang male	0.1621	0.1534	0.1335	0.0000		
‘Dongkui’ female series	0.2066	0.2085	0.1910	0.2346	0.0000	
Mixed male	0.0876	0.1040	0.0849	0.0728	0.1919	0.0000

### Genetic relationship among the accessions

**Fig. 4:**
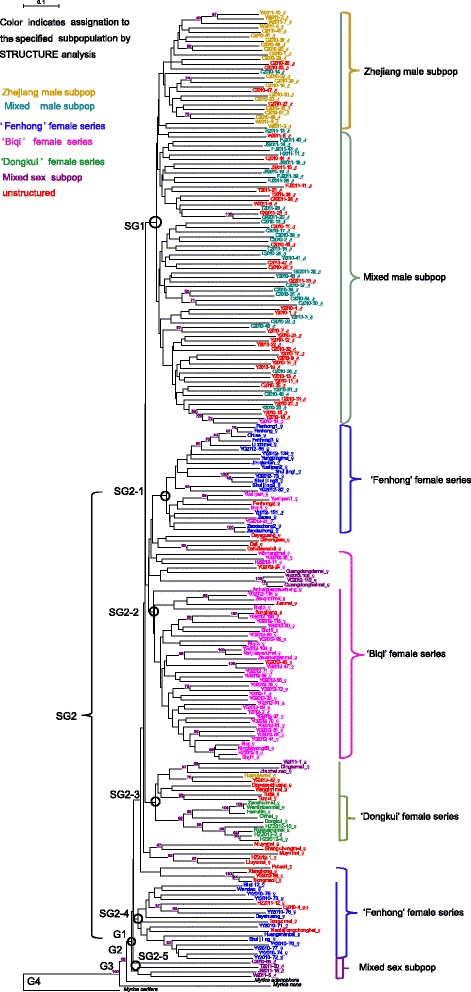
Neighbour-joining tree for the 216 *Myrica* accessions based on 84 SSRs. The tree was rooted using the related species ‘*Myrica cerifera*’ as outgroup. Bootstrap support values greater than 55 % are shown in blue on the branches. Subgroup numbers beside the tree nodes indicate the two groups, and SG2 can be subdivided into five subgroups. The sex of the accession is after the name. The subpopulation IDs are noted on the right. Accessions in different colour indicate they were assigned to corresponding subpopulations. Unstructured accessions are in red

Using a neighbour-joining (NJ) tree enabled a clear division of the 216 accessions into four main groups: G1, G2, G3 and G4 (Fig. 4). Three related species *M. adenophora*, *M. nana*, and *M. cerifera* fell into the G2, G3 and G4 groups, respectively, as outgroups. G1 contained all the *M. rubra* accessions, and can be divided into two subgroups: SG1 and SG2. Ninety-one male plants were assigned to SG1, and five major subgroups clustered in SG2, assigned 101 accessions and clustered according to the sex type and geographical origin in the dendrogram, also consistent with the structure-based membership assignment. The other accessions clustered into several small groups, with males and females assigned to distinct groups. Of the 98 male accessions, 91 were assigned to male group SG1, four to SG2-5, and three to the female groups, one to SG2-1, and two to SG2-2. ‘W2011-1’, collected from a female plant with male inflorescences, was close to ‘Dingaomei’ in SG2-3. The two cultivars ‘Biqi’ and ‘Dongkui’, and the landrace ‘Fenhong’, were split into different subgroups, suggesting a different origin.

The AMOVA and PCoA of six subgroups, based on the phylogenetic tree, were analysed. AMOVA analysis detected a variation of 79.01 % within individuals, while 10.97 % of the variation was attributed in six subgroups (Additional file 3: Table S2). The overall Fst in the six subgroups was 0.10972 (p value < 0.05) (Additional file 4: Table S3). A PCoA plot showed that the first and second coordinates accounted for 26.78 % and 20.03 % of the molecular variation, respectively. The first coordinate clearly separated SG1 (male accessions) from SG2 (female accessions), and the second coordinate separated SG2-2 from SG2-1, while the subgroups SG2-1 and SG2-3 could not be separated from each other (Additional file 5: Figure S2).

### Sex associated marker

**Fig. 5:**
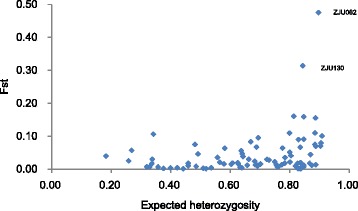
Distribution of Fst values from the 84 SSRs in function of the expected heterozygosity using FDIST2 software. ZJU062 and ZJU130 were identified as outlier loci above the 95 % confidence level

**Fig. 6:**
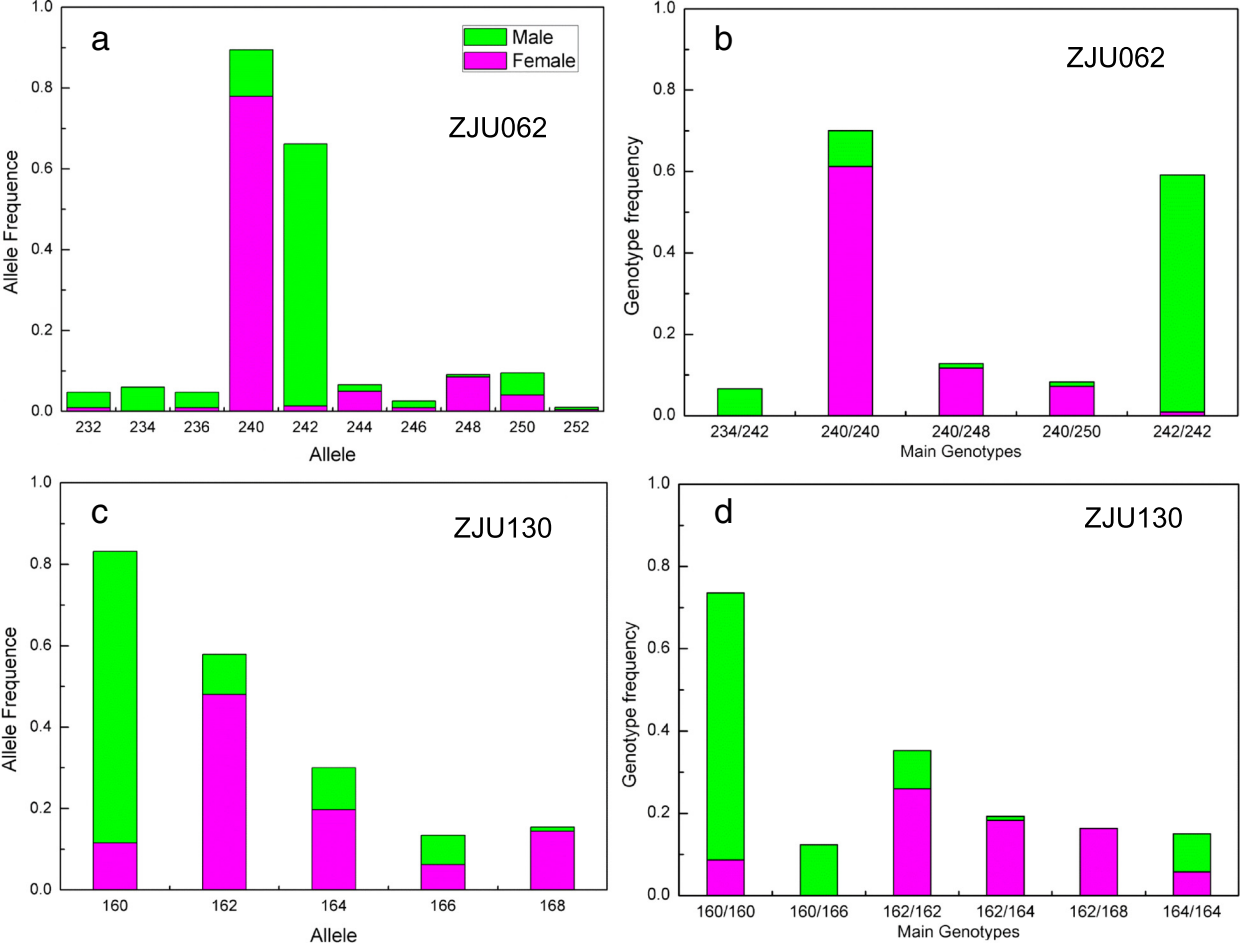
Allele frequency and main genotype frequency at SSR locus outliers. **a**, The allele frequency of locus ZJU062; **b**, Main genotype frequency of locus ZJU062; **c**, The allele frequency of locus ZJU130; **d**, Main genotype frequency of locus ZJU130. Note: Main genotype frequency is a genotype frequency above 0.05

To detect signatures of sex associated markers, we scanned the 84 SSR loci in both the male and female populations. SSR loci ZJU062 and ZJU130 showed Fst values (0.455 and 0.333, respectively) significantly above the 95 % confidence level by FDIST2 analysis (Fig. 5), indicating they are outlier loci probably related to sex separation. With a more detailed analysis of the allele frequency and main genotype frequency (>0.05) of these two loci in the populations (Fig. 6), we found that ZJU062-240 bp frequency was 0.774 and 0.133 in the female and male populations, respectively, while for ZJU062-242 bp this was 0.013 and 0.607 (Fig. 6a), respectively. Moreover, the frequency of 240/240 and 242/242 genotypes in the two populations was distinct (Fig. 6b). Similarly for the ZJU103 locus, ZJU130-160 bp was 0.119 in the female and 0.719 in the male population, while ZJU130-162 bp was 0.487 and 0.097, respectively (Fig. 6c). The main genotype of ZJU130 in the male population was 160/160 bp, while in the female population they were 162/162, 162/164 and 162/168 (Fig. 6d).

When we exclude the two SSR markers associated with sex, the phylogenetic tree (Additional file 6: Figure S3) showed that a small group of the male and female accessions were mixed together, not separated clearly as in Fig. 4.

## Discussion

### SSR polymorphism and genetic diversity

Here we studied the variability of a heterogeneous collection of 213 Chinese bayberry genotypes. The 99 male individuals were collected from five provinces of south China, with more than two thirds from six cities in Zhejiang Province. In total, the 84 polymorphic SSRs amplified an average of 10.43 alleles per locus, 26.9 % of which were rare alleles, higher than the value observed in previous studies [6,13,15]. These high values can be explained by a larger sample size and high heterogeneity of the sample.

The female and male accessions contributed equally to the variability, with a mean gene diversity value, *He*, of 0.65 for the 216 individuals, which suggested the rich genetic diversity of *M. rubra*. This was similar to that (0.66) reported by Xie, where 123 cultivars were analysed with 14 SSRs [6], and slightly lower than the figure of 0.72 estimated by Jiao, who studied 23 landraces and six male accessions of *M. rubra* [13]. Heterozygosity of the females was slightly higher than that of the males (0.65 versus 0.62) but the difference was not significant, in part due to the wide genetic background in the samples and to the cross pollination in dioecious plants. Some of the landraces also clustered within their own gene pools, especially those from Zhejiang Province: ‘Biqi’, ‘Fenhong’ and ‘Dongkui’. Based on our results, it is clear that germplasm from Zhejiang Province, with its rich genetic diversity, should be widely used in breeding programs.

### Population structure

Bayesian clustering analysis has been proven to be powerful tools to evaluate the genetic structure of fruit tree populations. A nested clustering strategy has also been used to assign individuals to populations, for example in apple [27] and peach [18]. The primary genetic separation in our data divided the male and female collections into two subpopulations, with a few exceptions, with low genetic structure suggesting that gene flow between them is high. In the STRUCTURE analysis, 82 males and 16 females were assigned to the Male-dominated population, which can be further clustered into four subpopulations (Fig. 2b), suggesting a mixed genetic background of both male and female groups. The population differentiation value between the ‘Dongkui’ female series (close to the male subpopulation) and the two female subpopulations (‘Biqi’ and ‘Fenhong’) indicates early differentiation of the ‘Dongkui’ female from the males. This can explain why, applying growth regulator to control vegetative vigour, the ‘Dongkui’ cultivar is prone to bear male flowers [28], as well as provide evidence that male and female of Chinese bayberry may share common ancestors and co-evolved. Dioecious plants may have evolved from hermaphrodite ancestors [29]. The lower population differentiation value found between the ‘Zhejiang male’ and ‘Mixed male’ subpopulations suggests that sex differentiation occurred earlier than genetic differentiation.

Also using STRUCTURE as the first step, most Zhejiang landraces clustered together (Female Dominated pop., Fig. 2a). However, further analysis separated this cluster into two subpopulations, the ‘Biqi’ female series and the ‘Fenhong’ female series (Fig. 2c), with a low population differentiation value between Zhejiang landraces, revealing different germplasm sources in Zhejiang Province. Archaeological and written evidence both suggest that bayberry existed in Zhejiang Province 7,000 years ago, in Hemudu (Yuyao, Zhejiang Province), and was widely cultivated and grafted in Zhejiang Province with elite cultivars introduced into other provinces of China since the Southern Song Dynasty (1127–1279) [1]. This suggested that Zhejiang would be the centre of diversity of *M. rubra*. ‘Biqi’ and ‘Fenhong’ would be old landraces, possibly sharing one or a number of common ancestors. However, this remains to be further elucidated.

### Phylogenetic clusters

Phylogenetic analysis grouped *M. rubra* accessions into two subgroups consistent with sex type and geographic origin. The results were also in agreement with population structure and PCoA analysis. Collections from Fujian Provinces (Anhaipianzaosheng and Zaoshenganmei) are clustered with those from Zhejiang Provinces (Zaoqimimei and Sanjiaaowumei) in SG2-2, indicating gene flow occurred frequently among these two provinces [5,6]. Zhejiang has a long history of bayberry cultivation, and the excellent cultivars have been spread to surrounding provinces [1]. The landraces, ‘Fenhong’, and two best-known main cultivars ‘Biqi’ and ‘Dongkui’, were assigned to the subgroups SG2-1, SG2-2 and SG2-3, respectively, indicative of the rich germplasm in Zhejiang Province. The relatively long genetic distance between ‘Shuijing’ and ‘Shuijing2’ in SG2-1 is probably because ‘Shuijing’ was not a single but a group of cultivars [17], a phenomenon also found in ‘Fenhong’, ‘Yuelipan’ (SG2-1) and ‘Biqi’ (SG2-2). The Taiwan and Japanese cultivars, ‘Heiruilin’ and ‘Ruiguangmei’, clustered within SG2-3 and were closely related to cultivars from Zhejiang Province, consistent with the STRUCTURE results and confirming previous findings [5,6,17].

In general, males from geographically closer locations had higher genetic similarities than those from more distant locations, however, the three male plants of Y2012-1, Y2012-2 and Y2012-151 were clustered in SG2-1 and SG2-2, close to female plants collected from the same location, which indicates that these plants may be progeny of the female plants. Though few male accessions are clustered with female accessions, we would speculate that the male and female populations essentially have similar genetic diversity but distinct background associated with sex.

### Sex determination in *M. rubra*

Dioecious plants with separate male and female individuals are found in only 6 % of the 240,000 angiosperm species, and probably evolved recently from hermaphrodite ancestors [30]. Myricaceae probably evolved from polygamism through monoecism to dioecism [9]. Clarification of the mechanism for sex determination is important for developmental biology and breeding practices, but studies in dioecious plants have been limited to a few model plants, such as white campion (*Melandrium album*) [31-33] and *Rumex acetosa* [34,35]. Recently, in the study of papaya, bacterial artificial chromosome (BAC) libraries were constructed by sequencing the sex chromosomes [36], and then the X and hermaphrodite-specific region of the Y^h^ chromosome (HSY) were sequenced, with the results supporting the model of early sex chromosome evolution [37]. Sex determination and the sex chromosome of *M. rubra* and other related species have not been reported [9]. The SSR loci ZJU062 and ZJU130 were shown to be outlier (Fig. 5), so appear to be associated with sex segregation, However, excluding the two sex-associated SSR markers, the majority of female and male accessions were still distinct (Figure S3). Linkage status of these two SSR loci is under investigation. More research is needed to localize the precise genetic and genome positions controlling sex traits using segregating and natural populations of both sexes, screening with more SSR markers developed recently [13,16,17].

## Conclusions

Analysing male and female genotype data, we were able to estimate the genetic diversity of Chinese bayberry and demonstrate the rich diversity of the population. Phylogenetic cluster and population structure analyses revealed the sex trait effect on genetic structure stratification, and that Zhejiang Province could be the centre of diversity of *M. rubra*. The genetic diversity of the two cultivars ‘Biqi’ and ‘Dongkui’ and the landrace ‘Fenhong’ makes them an excellent source of variability for Chinese bayberry breeding programs. We also identified two SSR markers putatively associated to sex segregation in the two populations.

## Materials and methods

### Plant materials and tissue sampling

A total of 213 individuals of Chinese bayberry (*M. rubra*) and three related species (*M. adenophora, M. nana, M. cerifera*) were sampled. For *M. rubra*, 113 females, 98 males, one monoecious plant (C2010-4), as well as W2011-1 from a female plant with a male branch were used to evaluate genetic diversity and population structure. Among these collections, the male population was collected from five provinces in China, and half of the Yuyao and Cixi accessions from trees planted in avenues located in different areas of Zhejiang Province, with these male trees collected from different regions in Ningbo, Zhejiang Province. Of the female population, 34 were from the China Bayberry Germplasm Repository (CBGR), Yuyao, Zhejiang Province, China, and 62 were local landraces (Additional file 7: Table S4; Additional file 8: Figure S4). We used the Juno SC handheld (Trimble) GPS to obtain the geographic position of each individual. Among the 216 accessions, for 32, genotypic data was obtained from previous reports by Jiao [13], and the genotype of the three accessions ‘Biqi’, ‘Dongkui’ and ‘Fenhong’ were used as amplified PCR band size control.

### DNA extraction

Young leaves were collected from healthy trees, frozen in liquid nitrogen, and then stored at −40 °С prior to DNA extraction. The genomic DNA was extracted by the optimized CTAB method as previously described [5], and the DNA was quantified in an ultraviolet spectrophotometer (Eppendorf) and diluted to 20 ng/μl for PCR amplification.

### Microsatellite marker amplification

A total of 84 primers pairs (Additional file 1: Table S1) were selected according to their high polymorphism in previous studies: six EST-SSR markers with MRU/MYB as prefix were developed from the *M. rubra* EST database [15], and the remaining 78 were genomic SSR markers [13,14]. Forward primers were labelled with different fluorescence: NED, PET, FAM and HEX (Invitrogen). PCR amplifications were performed in an Eppendorf Mastercycler (Germany), with amplification reactions and temperature cycles according to the protocol described by Terakawa et al., Zhang et al., and Jiao et al. [13-15]. PCR products were checked on 1 % agarose gel at 100 V (m · v^−1^), and PCR products, with four different colour labelling, were then mixed with the internal size standard LIZ500 (ABI) and separated by capillary electrophoresis in an ABI 3130 Genetic Analyzer (Applied Biosystems, Foster City, CA, USA). Allele size was estimated with the GeneMapper v4.0 software (Applied Biosystems, Foster City, CA, USA). The genotypes of 216 accessions were deposited in Dryad (doi: 10.5061/dryad.7qc88).

### Data analysis

#### Analysis of genetic diversity

SSR data were scored as two alleles per locus distinguished by their size. We did not found multi-locus cases. The number of alleles (*Na*), the effective number of alleles (*Ne*), observed heterozygosity (*Ho*), expected heterozygosity (*He*), genetic diversity (Hs), Shannon’s information index (*I*) and Wright’s fixation index (F = 1-Ho/He) were calculated using GenAlEx 6.4 [38]. Since the sample size of the two population types was different, the number of distinct alleles and private alleles (not found in other populations) of the male and female population was estimated by ADZE [39], which employs a rare faction approach to obtain sample-size corrected estimates. Detection of ‘outlier’ loci was performed with FDIST2 software [40,41]. We simulated the neutral distribution of Fst with 50,000 interactions at the 99 % confidence level.

### Population structure

Genotype data from 213 *M.rubra* accessions were used to study population structure using the STRUCTURE v.2.0 software [42], employing an admixture model and correlated alleles frequencies. K values ranging from 1 to 10 were adopted to infer the number of clusters for ten replicate runs, with a 100,000 iterations burn-in period followed by 100,000 iterations MCMC (Markov chain Monte Carlo). The STRUCTURE HARVESTER web-based program was used to estimate the most appropriate K value [43], and CLUMPP (CLUster Matching and Permutation Program) software to calculate the average membership coefficient for each accession [44]. Accessions were assigned to a subpopulation when their membership coefficients were Q ≥ 0.6, and a bar-plot of the results prepared using DISTRUCT software [45]. Nested analysis of each of the subpopulations was using the same software and parameters as in the first STRUCTURE run.

### Analysis of molecular variance (AMOVA)

The genetic variation within and among different subpopulations and subgroups of Chinese bayberry accessions and pairwise Fst were calculated by analysis of molecular variance (AMOVA), using Arlequin v3.5 software [46]. Principal coordinate analysis (PCoA) was used to further confirm the cluster analysis results, using GenAlEx 6.4 [38].

### Phylogenetic analysis

Cluster analysis was conducted using the Neighbour-joining algorithm as implemented in TREECON ver.1.3 b [47]. Genetic similarity among all the accessions was estimated according to the Nei and Li genetic distance [48]. Bootstrap analysis was performed 1000 times to test the reliability of branches [49], and the tree was rooted using the related species ‘*M. cerifera*’ as outgroup.

## Additional files

Additional file 1: Table S1. Diversity parameters of the 213 Chinese bayberry accessions. *Na,* number of observed alleles; *Ne,* effective number of alleles; *Ho*, observed heterozygosity; *He*, expected heterozygosity; I, Shannon’s information index: Gn, number of genotypes at each locus. Additional file 2: Figure S1. The mean expected number of distinct and private alleles per locus as a function of standardized sample size for the male and female populations. Additional file 3: Table S2. Analysis of molecular variance (AMOVA) based on the 84 SSR loci of 192 *M. rubra* accessions among six major subgroups inferred from phylogenetic tree analysis (p < 0.05). Additional file 4: Table S3. Pairwise estimates of Fst based on 84 SSRs among the six major subgroups inferred from phylogenetic tree (p < 0.05). Additional file 5: Figure S2. Principal coordinate analysis (PCoA) of 192 *M. rubra* accessions. The different colours represent the six major subgroups inferred by phylogenetic analysis. The first and second principal coordinates account for 26.78 % and 20.03 % of the total variation, respectively. Additional file 6: Figure S3. Neighbour-joining tree for the 213 *M. rubra* accessions based on 82 SSRs. The font colour of the accession indicates the sex: red, male plant; black, female plant; blue, monoecious plant. The numbers are bootstrap values based on 1000 iterations. Only bootstrap values greater than 55 are indicated. Additional file 7: Table S4. The 213 bayberry accessions and three related species included in this study. ♀, female plant; ♂, male plant; ♀♂, monoecious plant. W2011-1 collected from female plant with one branch male mutation. Genotypes of two sex associated markers are included. Accessions belonging to different subpopulations are shown based on STRUCTURE analysis. Additional file 8: Figure S4. Map of China indicating the region of origin of the tree of the Chinese bayberry accessions.

## Abbreviations

CLUMPP: CLUster matching and permutation program
AMOVA: Analysis of molecular variance
PCoA: Principal coordinate analysis
FDIST: F distribution
