# Supplementary figures and images for: Genetic diversity of male and female Chinese bayberry (Myrica rubra) populations and identification of sex-associated markers
Source: BMC Genomics. 2015 May 19;16(1):394. doi: 10.1186/s12864-015-1602-5 (PMC4436740; doi:10.1186/s12864-015-1602-5)

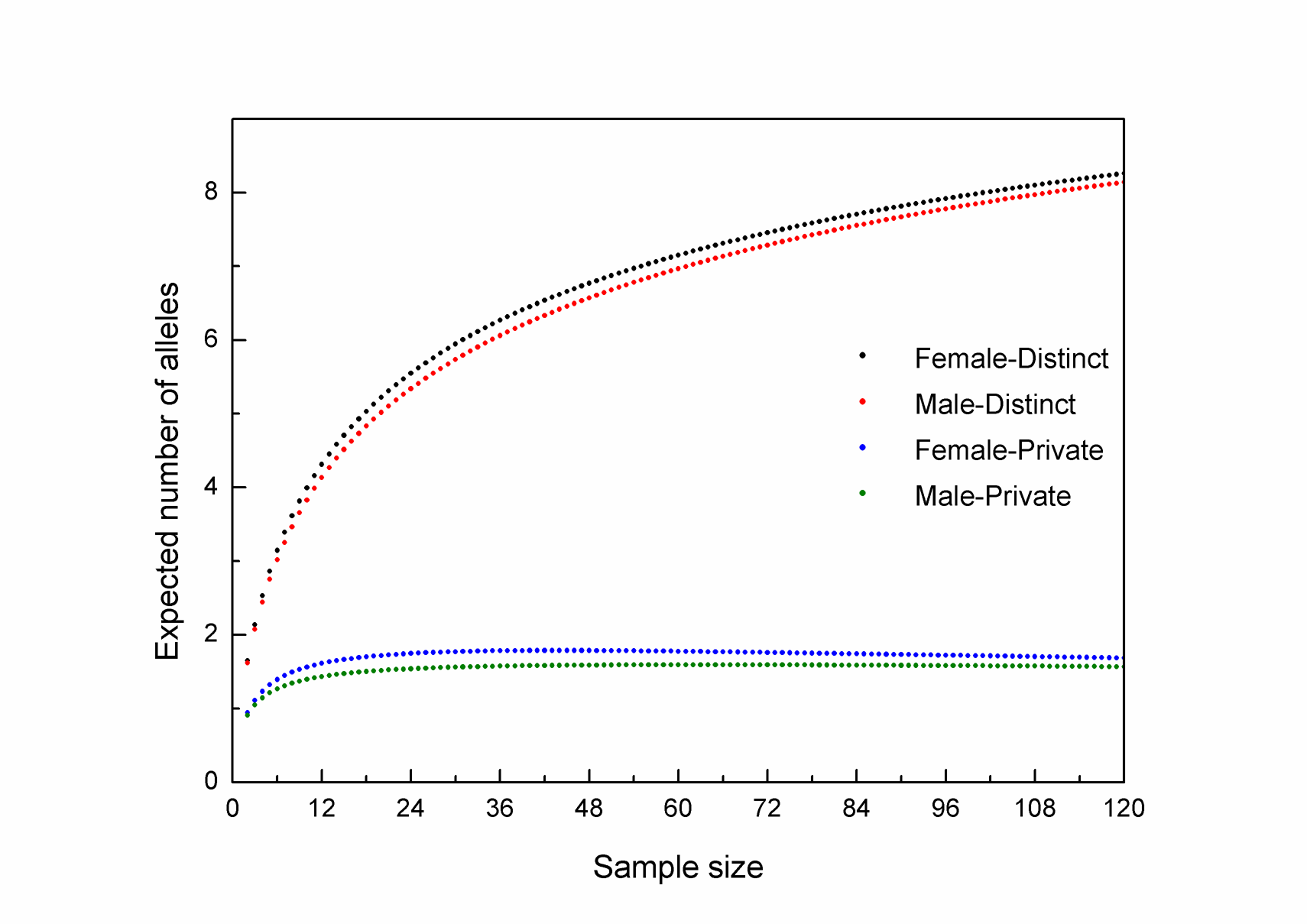

Supplement: Additional file 2: Figure S1. — The mean expected number of distinct and private alleles per locus as a function of standardized sample size for the male and female populations. [file 12864_2015_1602_MOESM2_ESM.tiff]

## Slide 1
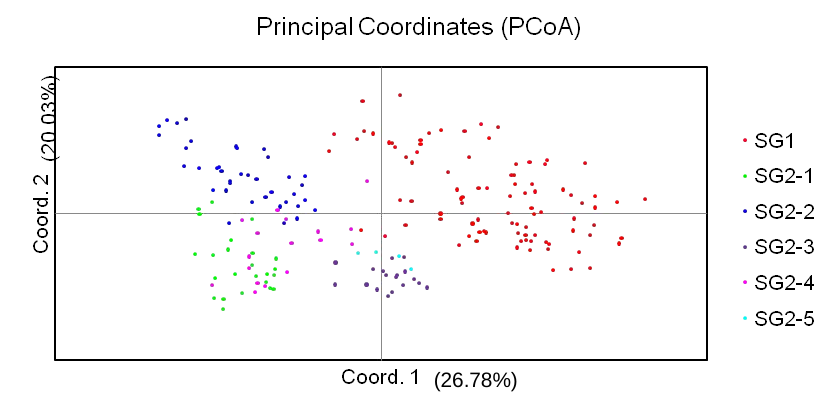

(26.78%)

Supplement: Additional file 5: Figure S2. — Principal coordinate analysis (PCoA) of 192 M. rubra accessions. The different colours represent the six major subgroups inferred by phylogenetic analysis. The first and second principal coordinates account for 26.78 % and 20.03 % of the total variation, respectively. [file 12864_2015_1602_MOESM5_ESM.ppt]

0.1

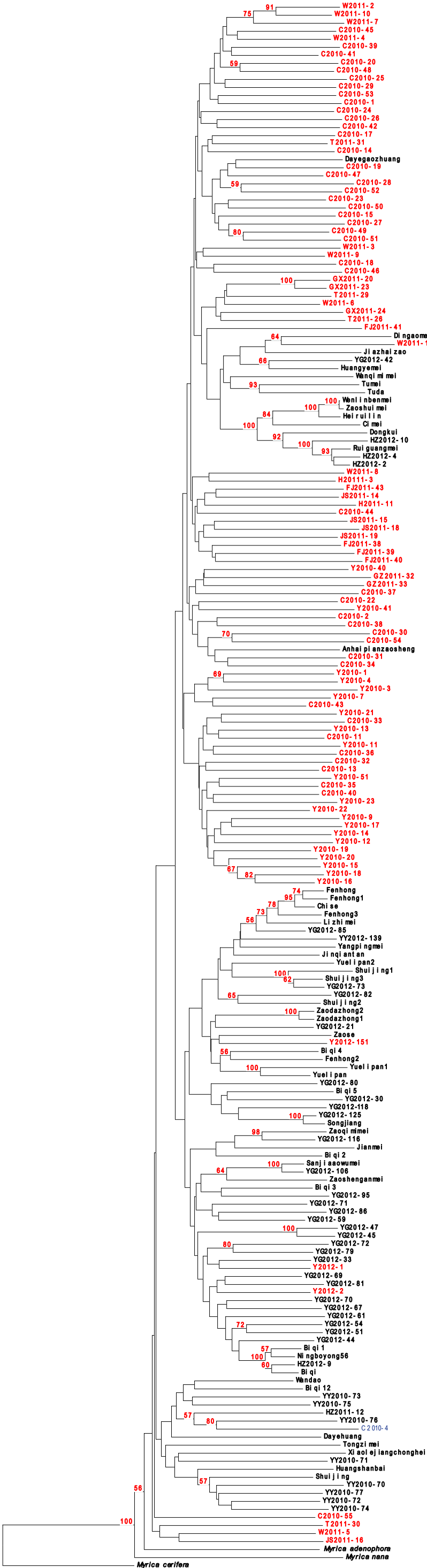

Supplement: Additional file 6: Figure S3. — Neighbour-joining tree for the 213 M. rubra accessions based on 82 SSRs. The font colour of the accession indicates the sex: red, male plant; black, female plant; blue, monoecious plant. The numbers are bootstrap values based on 1000 iterations. Only bootstrap values greater than 55 are indicated. [file 12864_2015_1602_MOESM6_ESM.pdf]

## Slide 1
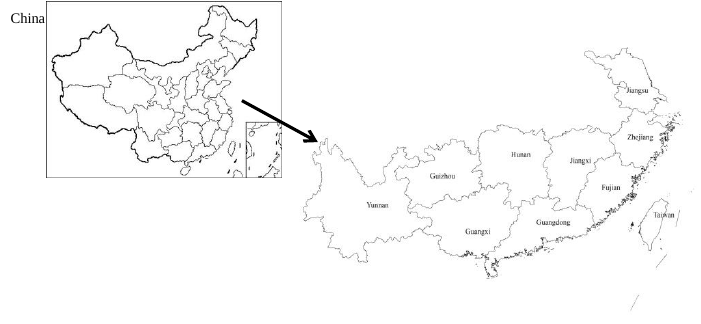

China

Supplement: Additional file 8: Figure S4. — Map of China indicating the region of origin of the tree of the Chinese bayberry accessions. [file 12864_2015_1602_MOESM8_ESM.pptx]
