# Supplementary material for: Genetic diversity of male and female Chinese bayberry (Myrica rubra) populations and identification of sex-associated markers
Source: BMC Genomics. 2015 May 19;16(1):394. doi: 10.1186/s12864-015-1602-5 (PMC4436740; doi:10.1186/s12864-015-1602-5)
Supplement: Additional file 3: Table S2. — Analysis of molecular variance (AMOVA) based on the 84 SSR loci of 192 M. rubra accessions among six major subgroups inferred from phylogenetic tree analysis (p < 0.05). [file 12864_2015_1602_MOESM3_ESM.docx]

**Analysis of molecular variance (AMOVA) based on the 84 SSR loci of 192 *M rubra* accessions within six major subgroups inferred from phylogenetic tree analysis.**

| **Source of variation** | **d.f.** | **Sum of squares** | **Variance components** | **Fixation Indices** | **Percentage of variation** |
| --- | --- | --- | --- | --- | --- |
| Among populations | 5 | 579.625 | 1.83843 Va | Fst=0.10972 | 10.97 |
| Among individuals within populations | 186 | 3086.875 | 1.67826 Vb | Fis=0.11250 | 10.02 |
| Within individuals | 192 | 2542.000 | 13.23958 Vc | Fit=0.20987 | 79.01 |
| Total | 383 | 6208.500 | 16.75627 |  |  |

Fis: inbreeding coefficient of subgroups, Fit: inbreeding coefficient in the total sample, Fst: genetic differentiation among subgroups
