# Supplementary material for: Genetic diversity of male and female Chinese bayberry (Myrica rubra) populations and identification of sex-associated markers
Source: BMC Genomics. 2015 May 19;16(1):394. doi: 10.1186/s12864-015-1602-5 (PMC4436740; doi:10.1186/s12864-015-1602-5)
Supplement: Additional file 4: Table S3. — Pairwise estimates of Fst based on 84 SSRs among the six major subgroups inferred from phylogenetic tree (p < 0.05). [file 12864_2015_1602_MOESM4_ESM.docx]

**Supplemental Table3. Pairwise estimates of Fst based on 84 SSRs within the six major subgroups inferred from phylogenetic tree analysis.**

|  | SG1 | SG2-1 | SG2-2 | SG2-3 | SG2-4 | SG2-5 |
| --- | --- | --- | --- | --- | --- | --- |
| SG1 | 0.0000 |  |  |  |  |  |
| SG2-1 | 0.1192 | 0.0000 |  |  |  |  |
| SG2-2 | 0.0900 | 0.1178 | 0.0000 |  |  |  |
| SG2-3 | 0.0944 | 0.1558 | 0.1301 | 0.0000 |  |  |
| SG2-5 | 0.1245 | 0.1150 | 0.1222 | 0.1528 | 0.0000 |  |
| SG2-5 | 0.0987 | 0.2011 | 0.1584 | 0.1189 | 0.0574 | 0.0000 |
